# Atypical Presentation of Thoracic Disc Herniation: Case Series and Review of the Literature

**DOI:** 10.1155/2013/621476

**Published:** 2013-04-04

**Authors:** Ali Shirzadi, Doniel Drazin, Sunil Jeswani, Leah Lovely, John Liu

**Affiliations:** Department of Neurosurgery, Cedars-Sinai Medical Center, 8631 West Third Street, Suite 800E, Los Angeles, CA 90048, USA

## Abstract

Modern imaging has revealed that thoracic disc herniation (TDH) has a prevalence of 11–37% in asymptomatic patients. Pain, sensory disturbances, myelopathy, and lower extremity weakness are the most common presenting symptoms, but other atypical extraspinal complaints, such as gastrointestinal or cardiopulmonary discomfort, may be reported. Our objective is to make providers familiar with TDH's atypical symptoms to help avoid potential serious consequences created by a delay in diagnosis. We report the cases of two patients who each presented with atypical extraspinal symptoms secondary to a TDH. One patient presented with a chronic history of nausea, emesis, and chest tightness and MRI showed a large right paramedian disc herniation at T7-8. A second patient reported chronic constipation, buttock and leg burning pain, gait instability, and urinary frequency; an MRI of his thoracic spine demonstrated a central disc herniation at T10-11. TDH can present with vague extraspinal symptoms and unfamiliarity with these symptoms can lead to misdiagnosis with progression of the disease and unnecessary diagnostic tests and medical procedures. Therefore, TDH should be included in the differential diagnosis of patients with negative gastrointestinal, genitourinary, and cardiopulmonary system basic studies.

## 1. Introduction

New developments in modern imaging have revealed that thoracic disc herniation (TDH) is no longer considered rare, with a prevalence of 11–37% in asymptomatic patients [1–4]. Symptomatic TDH has been noted to be one in a million per year [5, 6], accounting for  0.15–4% of disc herniations requiring surgical treatment [7–11]. It is most commonly seen in patients in their mid to late adult life with no significant difference in gender [8, 12]. Pain, sensory disturbances, myelopathy and lower extremity weakness are the most common presenting symptoms [13]; but other atypical extra-spinal complaints, such as gastrointestinal or cardiopulmonary discomfort may be reported [5, 14–23], thereby requiring extensive medical workup before diagnosis and treatment by a spinal surgeon. Familiarity with TDH's atypical symptoms is essential to avoid potential serious consequences created by a delay in diagnosis.

We report the cases of two patients who each presented with atypical symptoms secondary to a TDH. In each case, Magnetic Resonance Imaging (MRI) of the thoracic spine demonstrated disc herniation despite widely different and atypical symptoms: nausea, emesis, chest tightness, and subjective right lower extremity weakness in the first patient; constipation, buttock and leg burning pain, gait instability, and urinary frequency in the second patient. 

## 2. Case  1

A 49-year-old male presented with an 11-month history of nausea and emesis accompanied by occasional chest tightness. He had an extensive gastrointestinal (GI) workup during a 12-day hospital admission without any significant findings. Although the patient denied any major back or lower extremities pain, he complained of subjective right lower extremity weakness over the previous 2 months without any dysesthesias. His past medical history was consistent with diabetes, hypertension, and osteopenia. He denied any history of trauma, GI diseases, or changes in bowel, bladder, or sexual functions. 

**Figure 1:**
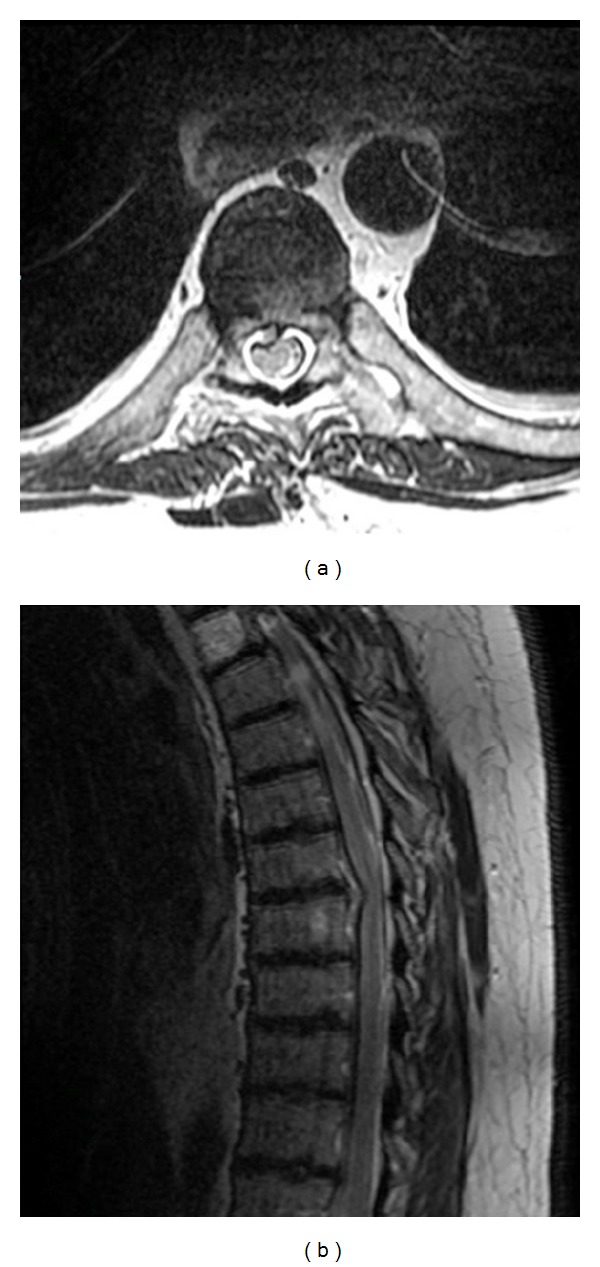
A thoracic spine T2-weighted axial (a) and sagittal (b). MRI reveals a right paramedian disc herniation at the T7-8 level with compression of the spinal cord. The disc appears to be soft and noncalcified.

On physical examination, the patient appeared in good health and was ambulatory without obvious deficits. His iliopsoas and quadriceps strengths on the right side were 2 and 4/5, respectively. Although he had diffuse diminished reflexes, no clonus was noted. His sensations were intact bilaterally. A thoracic spine MRI revealed a right paramedian disc herniation at the T7-8 level with compression of the spinal cord. The disc appeared to be soft and noncalcified. No myelomalacia changes in the cord were identified (Figure 1). 

The patient underwent a right-sided transpedicular approach to the T7-8 level with discectomy and decompression of the cord. Immediately after surgery, the patient's nausea improved and within 3 months his right iliopsoas and quadriceps strengths were improved to −4 and +4/5. Sixteen months after the operation, the patient is without discomfort or complaints.

## 3. Case  2

A 74-year-old male presented with buttock and leg burning pain, gait instability and weakness, constipation, and urinary frequency. These symptoms had been ongoing since 2008, however, had recently been worsening, especially his gait instability. 

A physical examination revealed full strength in both lower extremities with normal reflexes and no clonus or Babinski sign. He did seem to have an unsteady gait upon ambulation. No sensory level was present.

**Figure 2:**
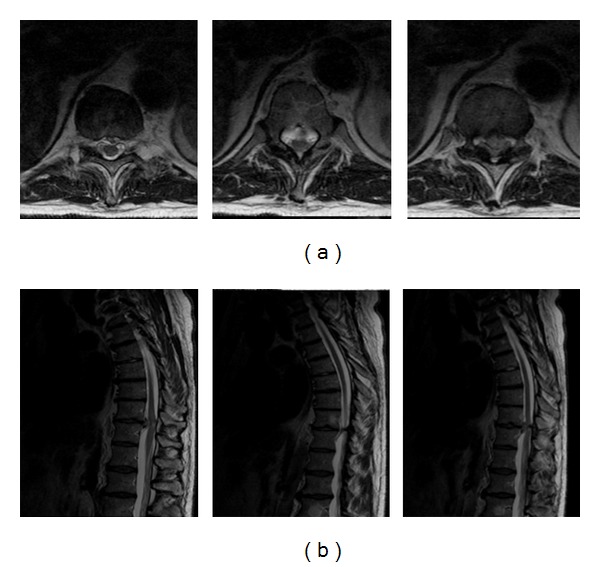
A thoracic spine T2-weighted axial (a) and sagittal (b). MRI showed a central disc herniation at the T10-11 level with compression of the spinal cord with myelomalacia changes at that level. The herniated disc appeared to be soft with no calcified component.

A thoracic spine MRI showed a central disc herniation at the T10-11 level with compression of the spinal cord with myelomalacia changes at that level (Figure 2). The herniated disc appeared to be soft with no calcified component.

Given the central location of the herniated disc, it was decided to take an anterolateral approach rather than a posterior approach. A double lumen endotracheal tube was placed by the anesthesiologist to allow deflation of the left lung, and the patient was placed in a right lateral decubitus position. After the incision, a rib was shingled and mobilized to allow for the transthoracic approach to the T10-11 disc space. Direct lateral tubes were docked onto the rib head of T11 with the assistance of dilators. Once the rib head of T11 articulating with the transverse process of T11 had been removed, a partial corpectomy of the posterior superior aspect of T11 and the posterior inferior aspect of T10 was performed. This allowed an adequate working corridor to either side of the midline at the disc space of interest. The herniated disc, as well as the posterior longitudinal ligament, was removed in fragments. Fluoroscopy verified the position past the midline, thereby confirming that adequate decompression had been achieved.

The patient did well postoperatively. His burning sensation in the lower extremities improved promptly after surgery. He was discharged in stable condition on postoperative day three. Now, fifteen months after the operation, the patient is neurologically intact and without any complaints.

## 4. Discussion

Since the first report of symptomatic TDH by Key in 1938, this rare pathology has challenged spinal surgeons [24]. Due to a low prevalence, a wide variety of clinical presentations, and the conflicting definition of “symptomatic disc,” the diagnosis and treatment of TDH is controversial [25]. Its low incidence is likely caused by the motion restriction of the thoracic anatomy due to the chest wall, smaller intervertebral discs, and dentate ligaments [19, 26, 27]. Disc herniation is most commonly noted in the lower thoracic spine with 75% of occurrences located below the 8th thoracic vertebrae [2, 28, 29], most likely due to greater mobility of the thoracic spine with increased exerted forces at these levels [2]. Cornips et al. associated these levels with an increased presentation of myelopathy due to increased mobility, delicate vascularization, and lack of a protective layer of peripheral white matter around the conus [28]. 

Foraminal disc herniation in the thoracic spine can lead to nerve root compression and severe chest or abdominal discomfort [19, 30–32]. Therefore, the clinical presentation may not correlate with the location of the herniation causing these patients to be frequently misdiagnosed by their primary physicians and leading to extensive, invasive, and expensive medical and surgical workups [33]. See Table 1 for a list of atypical presentations of TDH.

Although TDH can present with abdominal pain and vague symptoms of discomfort, the mechanism by which these symptoms occur is unclear. Intradural nerve roots from the lumbar enlargement to conus medullaris follow an organized pattern with most rostral roots being lateral [31]. Therefore, it has been speculated that higher more latter disc herniation could irritate the lower abdominal and pelvis roots and cause nonspecific discomfort. 

Additionally, visceral and somatic afferent fibers in dorsal columns, spinothalamic and spinocerebellar tracts, and dorsal and ventral horns have been recognized at different spinal levels [18, 20, 34]. Irritation of these tracts with their close association to the dorsal gray column of the spinal cord can cause referred pain and consequently, further abdominal discomfort [20]. Rohde and Kang proposed that compression of the cord at the site of visceral afferent fibers can lead to inflammation and hyperexcitability of visceral neurons [18]. This could possibly interfere with the descending inhibitory fibers that modulate noxious input leading to atypical presentations of TDH. Further research is required to explain these symptoms. 

Treatment of TDH may be determined by the patient's symptoms. Pain without further neurological abnormality can be managed conservatively with NSAIDs and foraminal steroidal/analgesic injections with a reported 75% success rate [8, 17, 35]. However, in patients presenting with myelopathy, intractable radiculopathy, or axial back pain, surgical decompression is advocated [10]. Surgical management of TDH has significantly evolved since the first attempt by Alfred Adson in 1922 [36, 37]. Although early studies with laminectomy were suboptimal with a 32% rate of neurological worsening [38, 39], safer approaches have been developed. These include the posterior (transpedicular and costotransversectomy), lateral extracavity, and anterior transthoracic (via thoracotomy or thoracoscopy) approaches to the thoracic spine. Fessler and Sturgill stated that the morbidity and mortality associated to these approaches are identical [39]. Thus, the proper approach should be based on (1) the anatomical location of the herniated material, (2) the general health of the patient, and (3) the surgeon's experience. Complication rates of 15–24% have been reported in the literature [13, 40, 41]. These include spinal cord injury, postoperative neuralgia, cerebrospinal fluid leak, and postsurgical kyphosis [38]. However, each approach has its own outlined risk. 

## 5. Conclusion

Thoracic disc herniation has a prevalence of 11–37% in asymptomatic patients and can present with vague atypical symptoms. These atypical symptoms make diagnosis challenging, which can therefore potentially lead to a misdiagnosis with progression of the disease. Familiarity with these atypical symptoms will assist providers in making a faster diagnosis and may help eliminate extensive, invasive, and expensive medical and surgical diagnostic workups. Therefore, TDH should be included in the differential diagnosis of patients with negative gastrointestinal, genitourinary, and cardiopulmonary system basic studies. 

## Figures and Tables

**Table 1:** Atypical symptoms attributable to thoracic disc herniation.

Atypical symptoms from thoracic disc herniation	
Constipation	
Hypotonic bladder	
Saddle anesthesia and/or lack of anal reflex	
Aconuresis	
Bowel dysfunction	
Emesis Gastroparesis	
Nausea	
